# Microfluidics for multiscale studies of biomolecular condensates

**DOI:** 10.1039/d2lc00622g

**Published:** 2022-09-21

**Authors:** Nadia A. Erkamp, Runzhang Qi, Timothy J. Welsh, Tuomas P. J. Knowles

**Affiliations:** Yusuf Hamied Department of Chemistry, Centre for Misfolding Diseases, University of Cambridge Lensfield Road Cambridge CB2 1EW UK tpjk2@cam.ac.uk; Cavendish Laboratory, Department of Physics, University of Cambridge J J Thomson Ave Cambridge CB3 0HE UK

## Abstract

Membraneless organelles formed through condensation of biomolecules in living cells have become the focus of sustained efforts to elucidate their mechanisms of formation and function. These condensates perform a range of vital functions in cells and are closely connected to key processes in functional and aberrant biology. Since these systems occupy a size scale intermediate between single proteins and conventional protein complexes on the one hand, and cellular length scales on the other hand, they have proved challenging to probe using conventional approaches from either protein science or cell biology. Additionally, condensate can form, solidify and perform functions on various time-scales. From a physical point of view, biomolecular condensates are colloidal soft matter systems, and microfluidic approaches, which originated in soft condensed matter research, have successfully been used to study biomolecular condensates. This review explores how microfluidics have aided condensate research into the thermodynamics, kinetics and other properties of condensates, by offering high-throughput and novel experimental setups.

## Abstract 2

Combining microfluidics with existing techniques allows for novel and more high-throughput measurements of the thermodynamics, kinetics and characteristics of biomolecular condensates.

## Graphical abstract

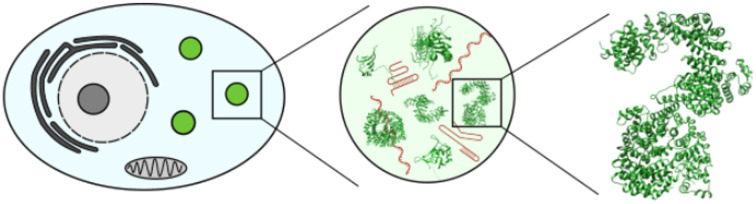

## 1 Introduction

**Fig. 1:**
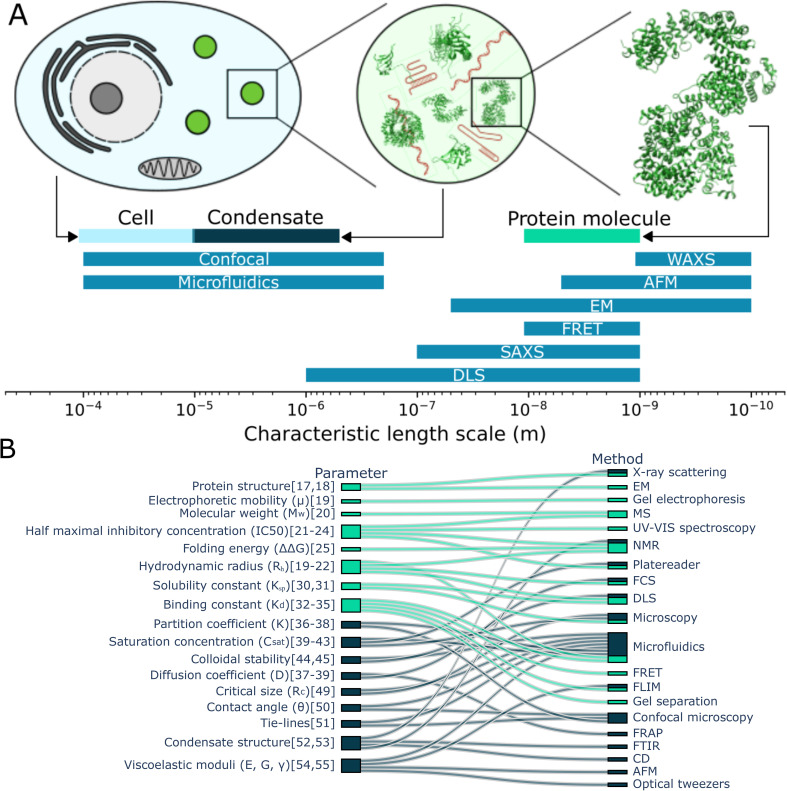
Characterisation of protein monomers and condensates. (A) Comparing the typical size of cells, condensates and proteins with the typical length-scale of measurement techniques, it becomes clear that some techniques used to study proteins are less suitable to study condensates. (B) The some of the parameters used to characterise protein monomers and their interactions (light blue) and condensates (dark blue) are connected with the (measurement) techniques to determine them. Studying monomers and condensates requires a range of techniques. Notably, studying condensates requires additional techniques in comparison to studying protein monomers and microfluidics can be used for together with many different techniques.^17–55^

Cells can reversibly form membraneless organelles, or biomolecular condensates out of proteins, nucleic acids and other small molecules (Fig. 1A). These condensates are capable of performing a range of healthy physiological functions such as, storage of nucleic acids,^1^ stress response,^2^ coordinating signalling pathways,^3,4^ regulating gene expression^5^ and selectively degradating RNA.^6^ Condensates form *via* a process called liquid–liquid phase separation (LLPS). In this process, proteins and nucleic acids homogeneously mixed in a solution spontaneously condense to form a distinct boundary between dense and dilute biomolecular phases.^7,8^ Often, the entropy cost of demixing is overcome by the enthalpy gain from the many interactions that are established between the biomolecules. These include cation–π, dipole–dipole, charge–charge and π–π stacking interactions.^9^ The biomolecules which make up condensates are typically multivalent, capable of sustaining multiple weak interactions,^10–12^ and partially intrinsically disordered, lacking a fixed 3-dimensional structure.^13^ Studying the mechanisms underlying condensate formation and maintenance as well as their function in cells is becoming increasingly more important to understand how these subcellular structures can dictate biological function and to understand their potential pathological effects.^14^

In contrast with studying proteins in solution, characterising condensates comes with a distinct set of challenges. Condensates are approximately 2–4 orders of magnitude larger than monomeric proteins, which makes some techniques used for studying proteins less suitable for studying condensates (Fig. 1A). On top of this, condensates have emergent properties; they are more than the sum of the proteins they are made off. For example, condensates can have a contact angle with surfaces, can sequester compounds and can obtain an internal structure, which is found when investigating the total structure rather than the separate proteins or even their interactions. In figure Fig. 1B, we consider the parameters used to characterise protein monomers and their interactions (aqua) and those to characterise condensates (dark blue). These parameters are connected to the techniques often used to aid in determining these values. We see that while some techniques can be used both for characterising condensates and monomers. However, we also see a significant difference in techniques and characterisation parameters, caused among others by the difference in length-scale and emergent properties of condensates.

**Fig. 2:**
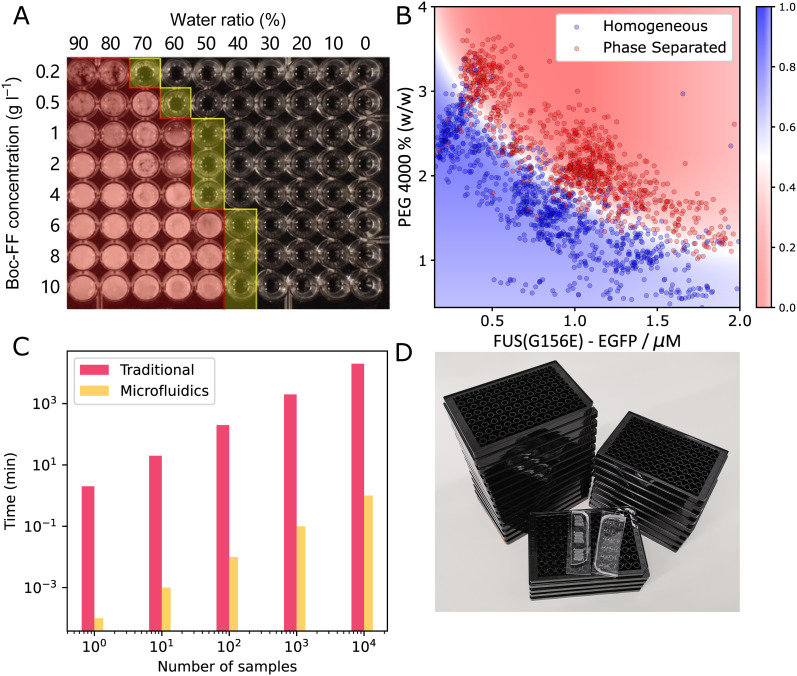
Comparing traditional method and microfluidics for phase diagram measurement. (A) A typical phase diagram generated by conventional platereader experiment. The water/ethanol ratio and Boc-FF peptide concentration was varied, yielding the rough boundary shown in yellow^15^ (B) a typical phase diagram generated using PhaseScan.^16^ (C) Comparison of the time consumption of traditional and microfluidic experiments for preparing phase diagrams (D) comparing the number of platereader plates (black) and microfluidic devices (transparent) needed to generate the amount of data points shown in (B). Fig. A and B are adapted from ref. 15 and 16 with permission.

From this overview, we can also see that microfluidics technologies are often combined with other techniques to determine a range of parameters when studying condensates. This includes the diffusion coefficient, saturation concentration of a phase transition, partition coefficient of multiple components into condensates and viscoelastic moduli. There are disadvantages to using microfluidics. It can be more technically challenging to set up initially and it might be more difficult to perform experiments with a large set of different compounds. However, using microfluidic methods often comes with great advantages, like being able to perform novel experiments and saving significant time and resources. Consider the preparation of a phase diagram by conventional pipetting methods. A protein stock solution is mixed manually with other solutions in a well plate. A platereader or microscope is used to determine the presence or absence of condensates. From this, a phase diagram is constructed such as seen in Fig. 2A.^15^ From this, a rough boundary between the phase separated and non-phase separated state can be determined (yellow). Each sample requires at least 1 μL of protein stock solution. Now, consider the process using microfluidic methods. Less than 20 pL of protein stock solution is required to make individual microdroplets, each of which represent a distinct point in concentration space. These microdroplets may be created and imaged in a highly multiplexed manner to create a robust phase diagram where a high resolution phase boundary may be directly extracted from the measured points (Fig. 2B^16^).

For further comparison, Fig. 2C shows the difference in time required to acquire datapoints for the phase diagram with (orange) and without (pink) microfluidics. Preparing phase diagrams takes 4 orders of mangnitude less time with microfluidics, which is particularly important when making phase diagrams with many datapoints. When preparing the phasediagram in Fig. 2B, you would need 25 platereader plates, or some PDMS microfluidic devices, as pictured in Fig. 2D. Thus, using microfluidics allows for more accurate phase diagrams and boundaries to be determined, when the same amount of material and time is available. This is only one example of the advantages of using small volume microfluidic methods in the study of biomolecular condensates and more will follow in the coming sections where we will discuss how microfluidics have so far assisted with characterising the thermodynamics, kinetics and other properties of condensates. We will also discuss what outstanding questions about biomolecular condensates remain to be answered.

## 2 Phase diagram of liquid–liquid phase separation

The phase diagram describes the phase behaviour of the system at equilibrium, from which valuable thermodynamic information can be extracted. In the context of liquid–liquid phase separation, the phase diagram is generated by determining the amount of phases when changing parameters such as concentration and property of the species, temperature, pH, solution, salt type and concentration, crowding agent^9,56^ and biomolecules such as ATP, RNA,^57^ DNA,^58^ molecular chaperon.^9,56^ Practically speaking, a sample is made by mixing protein, buffer and other molecules and then examined for the presence of condensates with methods including turbidity assays, which observes the existence of a second condensate phase, and centrifugation, which is often coupled with spectroscopy to determine the concentration of both the dense and dilute phases.^16,41,59,60^ As mentioned previously, a challenge for quantitative analysis of condensate phase diagrams is the limited amount of available date, because the measurement is not only labour intensive, but also sample-consuming, which is not affordable when characterising precious proteins. Therefore, the phase diagrams of proteins are usually characterised only to low resolution and for a limited set of proteins and conditions (Fig. 2). However, microfluidic techniques provides a unique opportunity for phase diagram measurements with low biomolecule consumption, low labour cost, fine resolution, and high throughput. In microfluidic systems, droplets with small amounts of materials can serve as independent micro-environments, to reduce material usage, massively parallelize and automate the measurement.

**Fig. 3:**
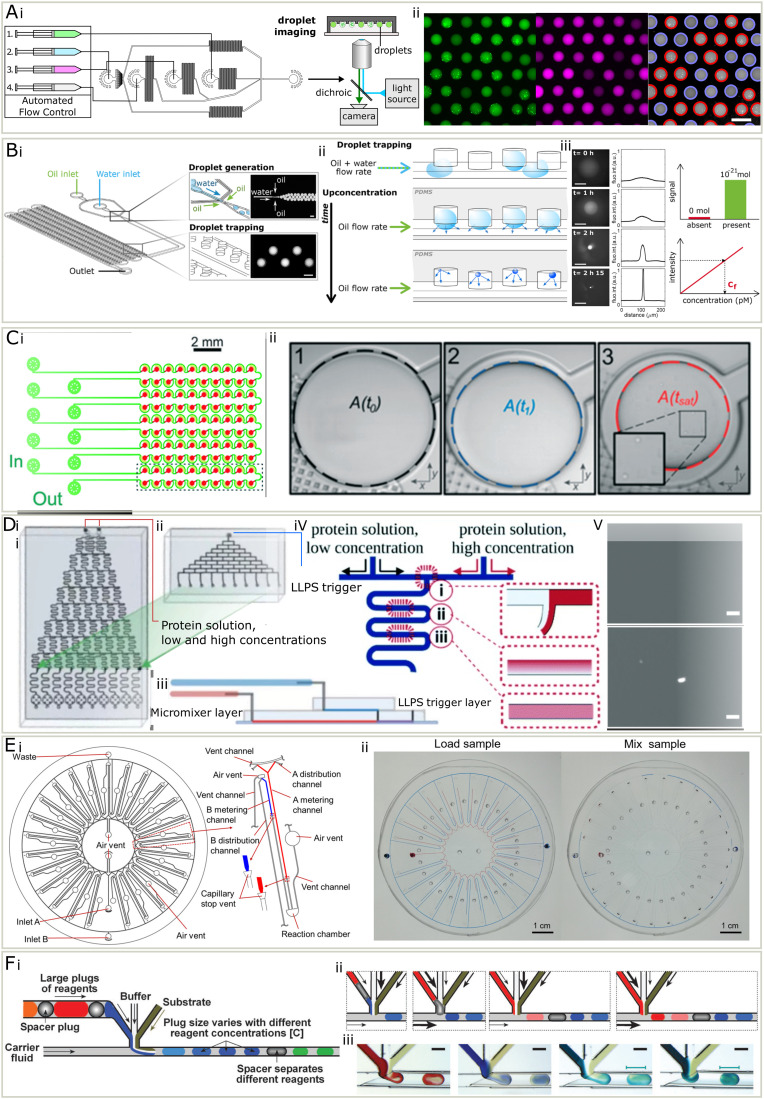
(A) PhaseScan^16^ (i) three inlet streams (1–3) were injected at varied flowrates, along with fluorinated oil (4), to generate droplets. The droplets were collected for fluorescent imaging. (ii) Left to right: Fluorescent micrographs in different channels and droplets were labelled in red if contained condensates and in blue if not. (B) The DroMiCo chip.^60^ (i) Device design. (ii) Droplets shrank over time and analytes concentration increased. (iii) As they shrank, the fluorescent intensity increased. Scale bar is 100 μm. Trace amount of materials could be measured due to low detection limit. The concentration calculated using calibration curve and intensity. (C) Phase diagram measurement chip with capillary valve.^41^ (i) It contained 5 units, each included 20 wells. (ii) The droplet shrank and the area when condensates appear was recorded to calculate concentrations. D A design of phase diagram measurement chip with serial dilution. (i) The chip used to create a concentration gradient across 10 outlets with two protein solution inputs with high and low concentrations. (ii) A top layer to combine with the chip shown in (i) for the injection of a LLPS trigger stream. (iii) The design of the complete microchip after stacking. (iv) A schematic for the diffusive gradient micromixer. (v) Top and bottom: Micrograph of a homogeneous state and LLPS respectively after mixing.^63^ (E) (i) A centrifugal microfluidic chip for phase diagrams, each containing 25 units (ii) when the sample was loaded, the difference in metering channel lengths leads to varied mixing ratio. Centrifugation forced the solutions into the reaction chamber to mix.^62^ (F) A design for droplet-based screening of rational crystallisation. (i) Schematics of droplet making with buffer, substrate and reagents in plugs. (ii) Schematics for generating droplets containing different reagents. (iii) Micrograph for generating droplets of different reagents.^64^ Adapted from ref. 16, 41, 60 and 62–64 with permission.

To date, several groups have presented microfluidic setups capable of measuring condensate phase diagrams. A study has reported a Monte-Carlo like method, where a library of droplets with varied concentrations of solutes were randomly generated, by injecting three sample streams at flow-profile-specified varied flowrates, along with fluorinated oil (Fig. 3A).^16^ The droplets were trapped and imaged with epifluorescent microscopy to extract the intensities of barcoding dyes premixed in sample solutions. Then a computer program was applied to analyse the image, both finding the concentrations of the samples inside each condensate by comparing with calibration series, and determining the phase behaviour inside each droplet. Each droplet corresponded to a data point on a phase diagram. In the phase diagram, a binodal could be determined by the boundary of the phase separated and homogenous regions.

Another method is to encapsulate materials in droplets and observe the first moment when condensate forms, while continuously removing the solvent over time. This can be achieved in several ways, including evaporation^60^ and osmosis.^61^ Additionally, researchers have reported a method to measure phase diagrams by gradually removing water *via* the PDMS environment (Fig. 3B).^60^ They designed a chip with flow focusing junction and wells made to generate and trap the droplets containing protein and other materials. A thin layer of PDMS sealed the wells from the outer environment but allowed the permeation of gas. The droplets lost water and shrank in volume over time, leading to the concentration of interior materials. Once the content was concentrated enough to form visible condensates, the volume of droplet was recorded to calculate the concentration of solutes. A similar method, but with capillary valves was also established.^41^ Droplets were generated by trapping aqueous phase in fluorinated oil primed wells, and removing excessive solution by filling with fluorinated oil (Fig. 3C). It does not require a syringe pump and is thus easier to operate. With this method, the accuracy, throughput and biomolecule consumption is largely improved compared to the conventional phase diagram characterisation approaches. The number of data points on the boundary can be easily increased by adding more droplet wells on the chip in parallel.

An alternative approach has been reported, which loaded and mixed small volumes of materials on a centrifugal chip and determine the phase boundary by sampling through a gradient of concentrations (Fig. 3D).^62^ Protein and another solution were injected to a disc-like microfluidic device, which contain 25 reaction units. Upon loading the sample onto the chip, the solution will enter metering channels and stopped by a capillary stop valve. Each reaction unit has different length of metering channels, corresponding to various mixing ratios. The microfluidic chip was then centrifuged so that the solutions would enter a reaction chamber. Inside the reaction chamber, from microscopic images, the status of the solution was determined and mapped to the phase diagram.

A study has proposed a method based on on-chip serial dilution and mixing (Fig. 3E).^63^ Two streams containing proteins in high and low concentrations were injected onto a microfluidic chip, and merged after a series of diffusive micromixer at varied ratio, leading to a concentration gradient across 10 flows. Then a LLPS trigger stream containing another molecule was introduced using another set of diffusive micromixers. From the following microscopy and the known mixing ratio, the resulting phase behaviour and corresponding conditions were determined.

Before the biological importance of condensates was widely accepted, microfluidics has been applied to scan the phase diagrams for protein crystallisation, for which similar setups can be used. These methods include valve-based microfluidics, droplet microfluidics, and slip chip. In valve-based chips, proteins and precipitants are loaded and mixed by the step-wise opening and closing of valves. In droplet microfluidics, proteins and precipitant solutions are mixed and encapsulated into tiny droplets isolated by surrounding inert oil in droplet-based microfluidics, which are made in varied concentrations by systematically changing the inlet flowrates.^42,64^ Slip chip is composed of two separate chips with wells. These can slip with respect to each other so that the reloaded samples in the wells contact and mix after motion, so that the phase behaviour of various mixing ratio and sample concentration combinations can be observed afterwards.^65^

A study^64^ reported a droplet-based method for protein crystallization, similar to PhaseScan,^16^ that can screen the conditions for multiple biomolecules in one experiment (Fig. 3E). This may point out a possible way that droplet-based phase diagram measurement can improve. In this method, the chemical space was screened by tuning the flowrates of three components with phase variations. What made it possible to screen multiple biomolecules is that the biomolecule inlet, instead of being one component, is composed of large plugs of biomolecule solutions in between spacer plugs (Fig. 3E). This idea may be implemented to phase diagram measurements and is not limited to screen different proteins, but also small molecules that may affect the phase behaviour of proteins in a highly automated manner.

In the future, the characterisation of phase diagram may evolve further to give more valuable information of a larger set of biomolecules at higher resolutions. It has been shown to be possible to measure multi-dimensional phase diagrams, which include multiple proteins and phase separation modulators, which may lead to better understanding of complex interactions of materials inside condensates, such as the synergistic effect of multiple components in condensates.^16^ These microfluidic-based methods may be extended and utilised to scan phase diagrams of spatial arrangement and morphology of condensates.^66^ Heating and cooling may be incorporated to measure phase diagrams at physiological and various temperatures, with pre-heated liquids, or Peltier stages.^67^ In addition to the saturation condition of protein, the concentration of materials in the condensate may be measured, potentially utilising confocal microscopy, fluorescence correlation spectroscopy, dynamic light scattering and volume fraction calculation. Lastly, more microfluidic setups developed for studying cells could be used to study condensates as well. Different environments can be created with high-throughput and precision using surface-wettability-guided assembly^68,69^ and microfluidic spraying.^70^

## 3 The kinetics of biomolecular condensates

Cells and their condensates operate out-of-equilibrium.^71^ Phase diagrams, which display the thermodynamic behaviour, therefore only provide part of the picture. In addition to thermodynamics, the kinetics of condensate formation is therefore another key question to understand their behaviour, and again microfluidics provide a distinct advantage in answering these questions due to the ability to work in small volumes, induce rapid mixing, and remove surface effects. Additionally, kinetic processes may be followed over short and long time scales.^72,73^ Kinetic studies of condensates have been performed in various different microfluidic setups.^9,74,75^ We will first discuss 3 setups used to study condensate assembly, disassembly, growth and ageing, before considering what questions about the kinetics of condensates still remain.

**Fig. 4:**
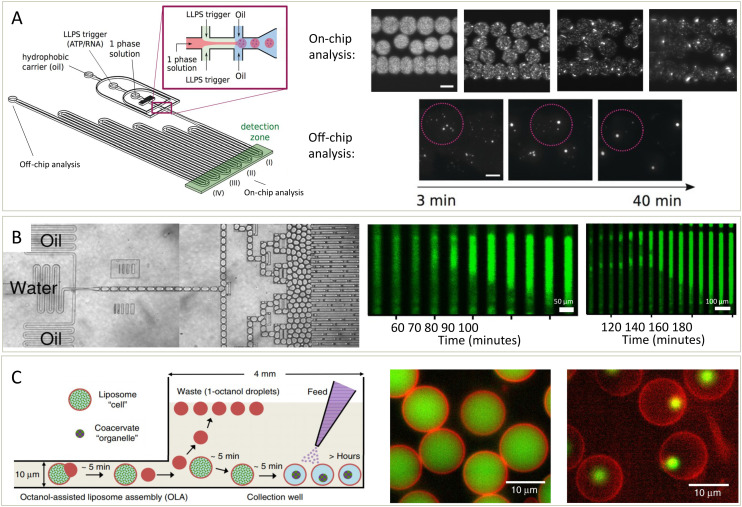
Platforms to study the kinetics of condensates assembling, growing, disassembling and solidifying. (A) Schematic overview of the droplet preparation and channel device (left).^49^ Droplets with condensates are made by mixing a protein (Dhh1) solution with a modulator, ATP and polyU, in oil. Over time, the formation and growth of condensates is followed (middle and right, scale bar is 100 μm). (B) Droplets containing protein, ThT and other compounds are prepared (left) and stored and imaged over time (middle and right).^76^ Fibrils nucleate in one (middle) or two (right) positions after which they nucleate throughout the droplet. (C) Schematic overview (left) of the formation of liposomes potentially containing condensates.^78^ Modulators influencing phase separation can either be present or be added later as feed to the liposomes, which have pores letting molecules move in or out. Based on the compounds added, the liposomes can contain a homogeneous solution (middle) or can contain condensates (right). Both the lipids (red) and condensate protein (green) are labelled. Adapted with permission from ref. 49, 76 and 78.

Microfluidics in combination with microscopy can be used to study the formation of condensates over time.^49^ The setup in Fig. 4A was used to study the phase separation of the DEAD-box ATPase Dhh1, an RNA-protein binding protein in P bodies, and how phase separation of this protein can be promoted by ATP and RNA. The protein was mixed with a modulator, ATP or RNA, in a microfluidic droplet, after which the distribution of protein in the droplets was studied over time. On the chip, the nucleation of condensates and how their size increased over time, mostly because of gravity-induced coalescence, was studied. Additionally, they could study droplets off chip on even longer times scales. The ability to simultaneously observe nucleation and growth kinetics in a single device is made possible only by the use of microfluidic droplet formation which allows condensates to be observed on a sufficiently long length scale.

In addition to studying nucleation and growth of condensates in a bulk droplet under laboratory conditions, questions remain on the effect of environment and spatial constraints on condensate dynamics. The setup described in Fig. 4B is similar to the setup in Fig. 4A in that proteins are mixed with other compounds in droplets and observed over time.^76^ However, this platform also allowed for the formed droplets to be incubated at different temperatures. At elevated temperatures, protein aggregation nucleated and spread through long droplets, either from one position (middle) or multiple position (right). Mixed into the droplets was thioflavin T (ThT), the intensity of which is a measure for the extend of aggregation.^77^

To study condensates is a biologically inspired system, microfluidics are used to form liposomes with pores (Fig. 4C).^78^ These pores allow exchange of material from outside and inside of the liposomes. Thus, phase separation could be induced, meaning that the liposomes went from containing a mixed solution (middle) to a phase separated system (right). After forming condensates inside of the liposomes, they were grown further by the addition of more protein or dissolved by the addition of modulators that inhibit phase separation. This allowed them to study the kinetics of these processes in a cell-like environment in exchange with its surroundings and this thus offers another setup for studying the kinetics of these processes.

Across the three methods mentioned, it is clear that microfluidic droplet technology is a key attribute in answering many biophysical questions regarding condensates. As has been shown, droplets allow for reactions to be highly multiplexed, sample volumes to be greatly saved, higher resolution datasets due an increase in number of assessed points over conventional techniques, and the ability to create a highly controlled environment where the surface interactions can be minimised.

The platforms described and similar platforms could be used to address remaining challenges about the kinetics of condensates. The solidification of condensates over time is very relevant for neurodegenerative diseases. However, many questions remain about how condensates solidify, form beta-sheets, become gel-like or form aggregates and particularly what effect environmental conditions, such as the presence of different compounds, concentrations and temperature, have on the length scales of the aggregation process.^39,79,80^ Beyond just their formation, there has been very little research into the internal structure of condensates. In cells, proteins and RNA can have a different concentrations in different places in the condensates. This heterogeneity has for example been observed in stress granules, which contain regions with more or less G3BP1 then other regions.^81^ This phenomenon is generally referred to as multiphase condensates. Besides this, there are also “hollow” or kinetically arrested phase separated condensates, which contain liquid with a low protein and low RNA concentration.^66,82^ Studies on the kinetics of the formation of multiphase and hollow condensates would help us to understand the origin and implications these structured condensates have in cells. The kinetics of the formation of these structures could potentially be studied in similar microfluidic devices as shown in Fig. 4. For example, the nucleation of an additional phase could be studied in similar fashion as the nucleation of the initial condensate phase. Additionally, more kinetic studies could be performed on condensates inside of cells, which are inside of microfluidic droplets. Factors like protein expression levels, posttranslational modifications, temperature and oxidative stress are known to effect the presence of condensates in cells.^83–85^ While internal structure plays a role in condensate behavior, the external factors in a cell which condensates can interact also dictate their behaviour. They can for example wet lipid membranes, similarly to how they can wet to other surfaces. There is still a lot unknown about what the effect of this wetting has on the ability to phase separate and solidify.^86^ In cells, condensates sequester components from the cell lysate. Microfluidics could help to perform high-throughput studies focused on the development of condensates for uptaking cargo molecules are sequestering chemical reactions. Understanding the kinetics of the sequestration and release processes will assist the development of these systems for bioengineering purposed of drug delivery and more.^36,87^ The microfluidics platforms discussed in this section, helped to better understand nucleation of condensates. To further improve our understanding, it would be of interest to determine growth rate and critical size of condensates.^88–90^ As these techniques develop, there are many exciting systems for which to kinetics remain a key questions. For example, a model system in which condensates can reversibly assemble and disassemble based on pH was developed^91^ and one where different kinds of FUS condensates can be obtained depending on heating.^92^ The kinetics of assembly and disassembly could be studied by mixing in solutions to change the pH or by changing the temperature. Using microfluidic setups, we could additionally improve our understanding of kinetics by studying the effect of crowder,^93^ ATP concentration^94,95^ and concentration of proteins.^96,97^

## 4 Characterization of condensate properties

In addition to understanding the kinetics and thermodynamics of condensates, the diverse nature of their building blocks – being made up of different proteins and nucleic acids of varying chemical composition, size, and compaction – gives rise to interesting properties with respective to their stability, charge, and material state. What makes these properties especially intriguing is that they are emergent, meaning they are not relevant for the monomer but become part of the system when larger assemblies form. Challenges persist in probing this aspects of condensate biophysics due to the influences of surface interactions on condensate assembly, and the micro-scale nature of condensates in general. Thus, microfluidic techniques have been developed to assess a handful of key properties of condensates, primarily their surface electrostatics, their ability to undergo a liquid-to-solid transformation under shear stress, and their rheological properties. It is the diverse nature of the molecules that make up condensate which make them have such unique characteristics ripe with challenges to address during their characterisation.

It is known that biomolecular condensates are typically made up of a mixture of proteins,^7,100^ nucleic acids,^1,101^ and other biological polymers.^102–104^ There are a range of molecules in condensates, but they often share the common property of being highly charged polyelectrolytes which underpins the fundamental process that many of these condensates are held together by a diverse set of electrostatic interactions, primarily charge–charge and cation–π interactions.^39,105^ Additionally, there are a suite of other non-ionic and hydrophobic interactions that hold condensates intact, yet even the non-charge driven interactions are heavily modified by the electrostatic environment in which LLPS occurs, such as the ability for high-salt environments to strengthen hydrophobic interactions.^106^ Not only has it been shown that electrostatic interactions are key for LLPS, but numerous papers have focused on how variation in charge–charge stoichiometry within condensates can influence their behaviour – including their morphology and their stability.^66,101,107^ This ability for electronic properties to modulate LLPS into biomolecular condensates is consistent with the finding that charged molecules (primarily nucleic acids) are key for the regulation of formation and dissolution of condensates *in vivo.*^101,108–110^ In addition to the typically studied micron-scale condensates, a new class of nanoscale condensates (100 nms in diameter) have been observed to form in sub-saturation conditions (*i.e.* below the boundary where large scale LLPS occurs),^111^ advances in microfluidic based detection has been key in the detection of these clusters in a non-size biased manner. These clusters are seemingly formed by tighter electrostatic interactions compared with the weaker and broader class of interactions, and point directly at the balance of strong and specific interactions *versus* weak more transient and non-specific interactions in regulation LLPS behavior. Since electrostatics are key in many area of biomolecular interactions^112^ is it no surprise that electrostatic interactions are relevant for LLPS across multiple length scales and they will continue to provide fodder for investigation for years to come.

**Fig. 5:**
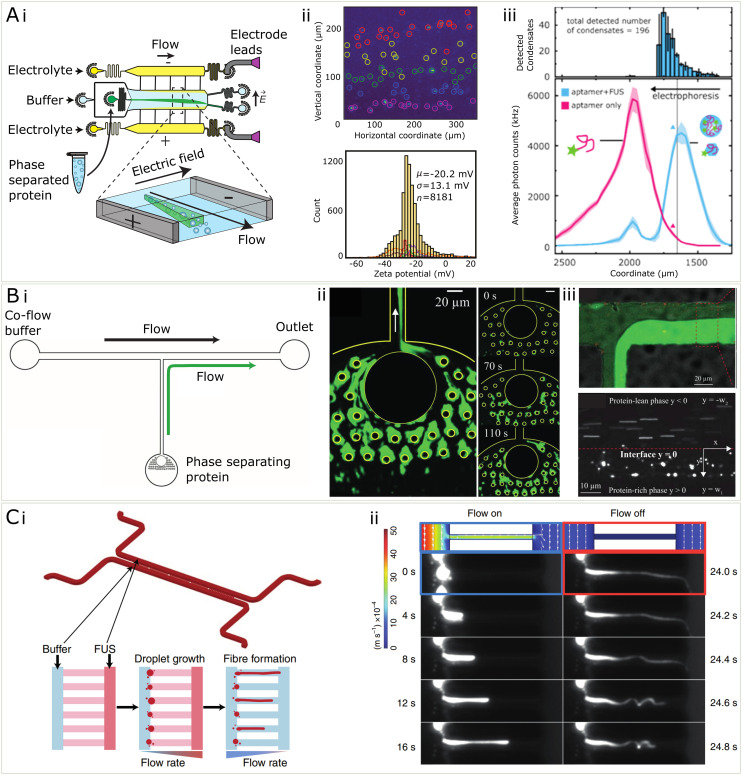
Microfluidic methods for the characterization of condensates (A) (i) μFFE is a microfluidic separation technique which is useful for the quantification of electrostatics properties of condensates (ii) μFFE may be used to characterize the zeta potential of condensates on the single particle level and assemble population distributions from samples of 1000 s of condensates^44^ (iii) Using single molecule counting and a fluorescent immunosensing probe, μFFE may be used for multidimensional characterization of condensates and binding events within condensates^98^ (B) (i) In order to measure the microrheology of condensates, they may be fused together in a microfluidic channel shown in i. (ii) Fluorescent microphotograph protein condensates stuck to PDMS pillars and coalesced. (iii) To measure viscosity, protein-rich phase and protein-lean phase were co-flowed and the velocity of probe beads were used to measure the viscosity of the condensate phase^99^ (C) (i) microfluidic device for measuring the relationship between shear stress and gelation of condensates (ii) by varying the flow rate within the channel, protein condensates were sheared to form fibres and a controlled pressure. The solid fibres recoiled when the flow was turned off, compared to the condensate only which did not.^54^

So far, the key contribution of microfluidics to better understanding the electronic properties has been the use of a technique called microfluidic free-flow electrophoresis (μFFE).^43,44,113^ μFFE is a method in which an analyte flows into a central microfluidic chamber where it is then subjected to an electric field perpendicular to the direction of flow (see Fig. 5Ai). The electric field comes from electrodes at the edge of the chamber which may be made of a low-melting point metal,^114^ or may be liquid electrodes of 3 M KCl solution under constant flow which ends up providing a more robust technique at higher voltages.^113^ This technique has been established for many uses in the analysis of biochemical mixtures, primarily it may be used for the quantification of protein charge and electrophoretic mobility, but also for analysing binding affinities between proteins and nucleic.^43^ In general, μFFE provides a microfluidic platform for determining electronic properties of an analyte while entirely immersed in solution and therefore minimising surface effects. Due to being an electrophoretic method, it is also applicable for separating out mixtures of proteins and nucleic acids, as has been previously done with streptavidin and DNA strands during the process of DNA aptamer development.^43^

In recent work,^44^ μFFE was used to quantify the zeta potential, or the electrokinetic potential at the edge of an electrolyte coat layer, of the surface of biomolecular condensates, on a single-condensate level. The main aim of this work was to resolve the heterogeneity and wide distributions of zeta potentials in a population of thousands of protein condensates (see Fig. 5Aii). This heterogeneity is attributed to the highly dynamic nature of these condensates, and their ability to adopt many different supramolecular geometries internally. Using μFFE on top of a standard epi-fluorescence microscope allows individual condensates (>200 nm in diameter) to be counted and thus thousands of individual point estimates for zeta potential may be found. Using microfluidics improves significantly over the typical method of dynamic light scattering (DLS) which is used to quantify zeta potentials of biomolecules and colloids in solution. The disadvantage of DLS is that the relative signal scales with the radius of the analyte to the power six. This means, for condensates, which vary greatly in size and composition, DLS provides a skewed estimate of zeta potential due to biasing the contribution from bigger condensates within the population.

μFFE was also used in the context of biomolecular condensates to assay binding affinities between RNA and FUS protein condensates,^98^ using the same microfluidic technique previously mentioned, instead mounted on a highly sensitive single-molecule counting confocal microscope which allowed for lower abundance samples (picomolar) to be quantified. In this usage, a low affinity RNA aptamer for FUS^115^ was added to preformed FUS condensates which resulted in a significant change in their electrophoretic mobility (see Fig. 5Aiii). Furthermore, the RNA was actually able to partially dissolve the condensates, a reentrant phase behaviour known to occur for monomeric FUS and RNA.^107^ This all allowed for three different peaks to be separated based on electrophoretic mobility, including free RNA, monomeric FUS bound with monomeric RNA, and full condensates associated with RNA. Because of the high separation resolution of the μFFE technique, binding affinities between the FUS and RNA and preferential partitioning of RNA into the condensates, as well as binding stoichiometries of the RNA in the condensate could all be quantified in a single measurement. Both of these uses of μFFE exhibit clearly how microfluidics provides increased resolution and ease of electrophoretic manipulation that make the measurements of fundamental properties possible within the heterogeneous and complex populations of biomolecular condensates.

While electrostatics are one force at play that dictates the assembly of condensates, the combination of various forces leads to varying levels of compaction, viscosity, and solidification. These parameters, among others, make up the set of material properties which are of interest for understanding condensate dynamics and behaviour, which is particularly relevant in understanding the role of condensates in disease^79^ and their potential uses for material fabrication. The study of material properties of a fluid are often measured using a large volume in bulk, however this is not possible in the case of biomolecular condensate studies since the material is too precious to be measured in large quantity. Luckily, in the past decades microfluidics has been extensively developed and become available for the manipulation and characterisation of materials at a small scale.^116–118^ In the future, some of these setups, originally developed to study cells, could be used to study biomolecular condensates as well.^119–121^ The low volume of microfluidic channels lead to reduced requirement for sample quantity. These properties have made microfluidics a promising platform for characterising the material properties of biomolecular condensates.

Microfluidics can be employed to measure rheological properties of condensates. Particle tracking is a widely used microrheology characterisation technique for studying the viscosity of biomolecular condensates.^5,48^ However, there is a lower limit of condensate for the technique to apply, because it requires the encapsulation of fluorescent particles in the condensates.^99^ As some biomolecular condensates are only in size of several micrometers, it might be challenging without further processing.^99^ Such a processing method has been developed which consisting of micro-scale pillars, which were capable of coalescing individual condensates into a large condensed phase, making such measurement feasible (Fig. 5A(ii)).^99^ In addition, the same method explored the possibility to apply real-time microrheology techniques of measuring viscosity by allowing the condensed phase to drip into a channel where it could be directly compared with a protein-lean phase co-flowed in a channel. Microbeads were trapped inside both phases and their velocity was tracked, which in combination with the known viscosity of the protein-lean phase, was used to calculate the viscosity of the condensed phase (Fig. 5Aiii).

Some condensates are prone to the change from the liquid to solid state, a process thought to be relevant in neurodegenerative diseases.^7,79^ Recently it has been more generally shown than a generic transition from liquid to solid state in possible for many protein and peptide condensate systems, including FUS, annexin A11, silk and zFF.^54^ To show this, condensates were grown in a microfluidic channel and shear stress was induced inside of a restriction channel by increased by changing flow rates to induce the aggregation process (Fig. 5(C)). Microfluidics allows for the a high level of control of the shear stress process on micron-scale condensates in a way not achievable by other methods, and can give insight on to how shear stresses that condensates feel when being transported around a cell may manifest into aggregation inducing shear. In addition to conventional methods that characterises the liquid or solid state of materials, including FRAP, microfluidics may be utilised to indicate the state of materials by the mechanical properties, such as elastic modulus. Although not yet applied to condensates, such methods have been explored in other studies, including cells,^122^ microgels^123^ and deformable particles.^124^ In these studies, external forces were applied to the object and the elastic modulus was calculated from the extent of deformation, generally microfluidic chips allowed for the controlled deformation of particles under specific pressure conditions induced by flowing fluids. This principle may be very useful in extending to the deformation of condensates within microfluidic devices to extract elastic and Young's moduli. These further insights provided by microfluidic studies of condensates allow us to understand their viscoelastic properties and how the mechanical environment which a condensate is in may influence its behaviour.

## 5 Conclusion and outlook

Biomolecular condensates perform cellular processes important for the cells functioning. It is thus important that we understand the mechanisms underlying the formation, maintenance and functions of these condensates and how they are related to diseases. Microfluidics are a very attractive tool to study condensates, since they allow for high-throughput study and manipulation of small samples over a wide range of time scales. When microfluidics are used to prepare phase diagrams, a significant reduction in time and material per sample can be achieved. Since, more data can be acquired, the effect of temperature, protein or nucleic acid concentration and crowder concentration on the ability of a system to phase separate can be investigated. In the future, studying the morphology of condensates, phase diagrams at different temperature and the concentration of other components in condensates would be of interest in order to make tighter ties between *in vitro* measurements and biology. Microfluidic setups have also been of great use to study the kinetics of condensates and shown for processes such as the formation and dissolution of condensates, which take place on a small time scale, can be studied as well as processes like the solidification of condensates, which take place on a longer time scale. Some future research directions include the internal structure of condensates, it's interactions with cell structures like lipid membranes and how condensates sequester compounds from the surrounding solution. In this review we have also discussed the importance of electrostatic interactions for the formation and function of condensates and shown how a single population condensates may contain condensates with varying composition and properties – thus microfluidic techniques with the ability to probe properties in a single-condensates yet high-throughput manner are key for assess the electrostatic nature of an entire condensate population. Microfluidics are very useful to measure the mechanical properties of condensates, which have a volume too low for most conventional measurement techniques. Techniques like coalescing condensates can help with this, but additionally, microfluidic setups which shear and squeeze condensates to find the Young's modulus, viscosity and surface tension have been developed. In addition to just understanding condensates, there is significant interest to engineer condensates for a range of functions from drug delivery to biomaterials. Microfluidic tools will continue to be paramount for the characterisation of novel condensates as they are continually engineered for new applications, and may also prove helpful for the sequential addition of components and the creation of microenvironments – both of which are easily possible within microfluidic droplets.

## Author contributions

N. A. E., R. Q., T. J. W. and T. P. J. K. wrote the original draft. N. A. E., R. Q., T. J. W. contributed equally to the paper. The co-first authorship order was determined by the website https://random.org.

## Conflicts of interest

The authors report no conflict of interest.

## Supplementary Material
